# Effects of coenzyme Q10 supplementation (300 mg/day) on antioxidation and anti-inflammation in coronary artery disease patients during statins therapy: a randomized, placebo-controlled trial

**DOI:** 10.1186/1475-2891-12-142

**Published:** 2013-11-06

**Authors:** Bor-Jen Lee, Yu-Fen Tseng, Chi-Hua Yen, Ping-Ting Lin

**Affiliations:** 1 The Intensive Care Unit, Taichung Veterans General Hospital, Taichung 40705, Taiwan; 2 School of Nutrition, Chung Shan Medical University, Taichung 40201, Taiwan; 3 Department of Family and Community Medicine, Chung Shan Medical University Hospital, Taichung 40201, Taiwan; 4 School of Medicine, Chung Shan Medical University, Taichung 40201, Taiwan; 5 Center for Education and Research on Geriatrics and Gerontology, Chung Shan Medical University, Taichung 40201, Taiwan; 6 Department of Nutrition, Chung Shan Medical University Hospital, Taichung 40201, Taiwan

**Keywords:** Coenzyme Q10, Statins, Antioxidation, Inflammation, Coronary artery disease

## Abstract

**Background:**

High oxidative stress and chronic inflammation can contribute to the pathogenesis of coronary artery disease (CAD). Coenzyme Q10 is an endogenous lipid-soluble antioxidant. Statins therapy can reduce the biosynthesis of coenzyme Q10. The purpose of this study was to investigate the effects of a coenzyme Q10 supplement (300 mg/d; 150 mg/b.i.d) on antioxidation and anti-inflammation in patients who have CAD during statins therapy.

**Methods:**

Patients who were identified by cardiac catheterization as having at least 50% stenosis of one major coronary artery and who were treated with statins for at least one month were enrolled in this study. The subjects (n = 51) were randomly assigned to the placebo (n = 24) and coenzyme Q10 groups (Q10-300 group, n = 27). The intervention was administered for 12 weeks. The concentrations of coenzyme Q10, vitamin E, antioxidant enzymes activities (superoxide dismutase, catalase, and glutathione peroxidase), and inflammatory markers [C-reactive protein (CRP), tumor necrosis factor-α (TNF-α), and interleukin-6 (IL-6)] were measured in the 42 subjects (placebo, n = 19; Q10-300, n = 23) who completed the study.

**Results:**

The levels of the plasma coenzyme Q10 (*P* < 0.001) and antioxidant enzymes activities (*P* < 0.05) were significantly higher after coenzyme Q10 supplementation. The levels of inflammatory markers (TNF-α, *P* = 0.039) were significantly lower after coenzyme Q10 supplementation. The subjects in the Q10-300 group had significantly higher vitamin E (*P* = 0.043) and the antioxidant enzymes activities (*P* < 0.05) than the placebo group at week 12. The level of plasma coenzyme Q10 was significantly positively correlated with vitamin E (*P* = 0.008) and antioxidant enzymes activities (*P* < 0.05) and was negatively correlated with TNF-α (*P* = 0.034) and IL-6 (*P* = 0.027) after coenzyme Q10 supplementation.

**Conclusion:**

Coenzyme Q10 supplementation at 300 mg/d significantly enhances antioxidant enzymes activities and lowers inflammation in patients who have CAD during statins therapy.

**Trial registration:**

Clinical Trials.gov Identifier: NCT01424761.

## Background

Cardiovascular disease is the leading cause of death worldwide [1]. Hyperlipidemia is a major risk factor for coronary artery disease (CAD). A higher level of low density lipoprotein-cholesterol (LDL-C) can increase the incidence of CAD [2]. The 3-hydroxy-3-methylglutaryl coenzyme A reductase (HMG-Co A reductase) inhibitors (statins) have been the most popular drugs for reducing the level of LDL-C, and they are an established strategy for decreasing the frequency of CAD events [3]. Coenzyme Q10 (also called ubiquinone) is a lipid-soluble benzoquinone that has 10 isoprenyl units in its side chain and is a key component of the mitochondrial respiratory chain for adenosine triphosphate synthesis [4,5]. Statins can decrease the synthesis of cholesterol and other molecules downstream of mevalonate. Mevalonate is a precursor of coenzyme Q10. Statins not only lower the blood cholesterol but also lower the level of coenzyme Q10 [6-9].

Higher levels of oxidative stress and inflammation play a role in the development of CAD [10,11]. Coenzyme Q10 is an intracellular antioxidant that protects the membrane phospholipids, mitochondrial membrane protein, and LDL-C from free radical-induced oxidative damage [12,13]. Recently, we have demonstrated that coenzyme Q10 had a cardio-protective impact on CAD. A higher level of plasma coenzyme Q10 (≥ 0.52 μmol/L) was significantly associated with a reduced the risk of CAD [14]. We proposed that a higher dose of coenzyme Q10 (> 150 mg/d) might show better antioxidation in patients who have CAD [15].

The circulating levels of C-reactive protein (CRP), tumor necrosis factor-α (TNF-α), and interleukin-6 (IL-6) are positively correlated with the risk of primary and recurrent myocardial infarction and death [16-18]. Adiponectin is an anti-inflammatory marker that is potentially antiatherogenic and is secreted in abundance by adipocytes [19]. Its level might be related to the development of CAD [20]. In a recent study [21], we reported that coenzyme Q10 administered at 150 mg/d decreased the inflammatory marker -IL-6 but had no effect on CRP in patients with CAD. Thus, we hypothesize that a higher dose of coenzyme Q10 (> 150 mg/d) would provide better anti-inflammation in CAD patients. The purpose of this study was to investigate the effect of coenzyme Q10 supplementation (300 mg/day, 150 mg/b.i.d) on antioxidant enzymes activities and anti-inflammation in patients with CAD during statins therapy.

## Methods

### Participants

This study was designed as a single blinded, randomized, parallel, placebo-controlled study. CAD patients were recruited from the cardiology clinic of Taichung Veterans General Hospital, which is a teaching hospital in central Taiwan. CAD was identified by cardiac catheterization as having at least 50% stenosis of one major coronary artery or receiving percutaneous transluminal coronary angioplasty (PTCA). The subjects in this study were treated with statins therapy for at least 1 month. The subjects with diabetes, liver, or renal diseases, or who currently use vitamin supplements were excluded. Informed consent was obtained from each subject. This study was approved by the Institutional Review Board of Taichung Veterans General Hospital, Taiwan.

With a sample size calculation, we expected that the change in the levels of antioxidant enzymes activities would be 5.0 ± 7.0 U/mg of protein after coenzyme Q10 supplementation; therefore, the desired power was set at 0.8 to detect a true fact and at an α value equal to 0.05 with a minimal sample of 18 in each intervention group. We enrolled 51 CAD patients in this study and used random numbers table to random assign the subjects to the placebo (n = 24) or to the coenzyme Q10 [300 mg/day (Q10-300 group), n = 27] groups. The female subjects in this study were postmenopausal women who were not receiving hormone therapy. The coenzyme Q10 and placebo (starch) capsules were commercially available preparations (New Health Taiwan Co., Ltd.). The intervention was administered for 12 weeks. The subjects were instructed to take two capsules daily (coenzyme Q10 supplements 300 mg/d, 150 mg/b.i.d). To monitor compliance, the researchers reminded subjects to check the capsules bag every 4 weeks to confirm that the bag was empty. The researcher also asked some questions to the subjects, such as ①When did you take the supplements? (We advised subjects to take the supplements after a meal); ②How many times did you take the supplements every day? (We advised subjects to take the supplements two times per day); ③How many capsules did you take of the supplements each time? (We advised the subjects to take one capsule of supplement each time); and ④How did you feel after taking the supplements? (for monitoring adverse effects), and then we checked the capsule bags. We placed 56 capsules in each bag, and the bag should be emptied after 4 weeks of taking the supplements. If the subjects took the empty bag back and answered the questions correctly, the researcher then gave them another bag of capsules. In addition, we also measured the concentration of coenzyme Q10 every 4 weeks supplementation for monitor the compliance of the subjects. The age, blood pressures, and smoking, drinking, and exercise habits of all of the subjects were recorded. The body weight and height were measured; the body mass index (BMI) was then calculated.

### Blood collection and biochemical measurements

Fasting venous blood samples (15 mL) were obtained to estimate the hematological and vitamin status. Blood specimens were collected in Vacutainer tubes (Becton Dickinson, Rutherford, NJ, USA) that contained EDTA as an anticoagulant or that contained no anticoagulant as required. Serum and plasma were prepared after centrifugation (3,000 rpm, 4°C, 15 minutes) and were then stored at -80°C until analysis. Hematological entities [serum creatinine, total cholesterol (TC), triglyceride, LDL-C, and high density lipoprotein-cholesterol (HDL-C)] were measured by an automated biochemical analyzer (Hitachi-7180E, Tokyo, Japan). The level of CRP was quantified by particle-enhanced immunonephelometry with an Image analyzer (Dade Behring, IL, USA). Plasma TNF-α (R&D Systems Inc., Minneapolis, USA), IL-6 (eBioscience, CA, USA), and adiponectin (BioVendor, Brno, Czech Republic) levels were measured by enzyme-linked immunosorbent assay (ELISA) using commercially available kits and according to the instructions made available from the suppliers.

Plasma coenzyme Q10 and vitamin E were measured by high-performance liquid chromatography (HPLC) and were detected by a UV detector at 275 nm and 292 nm, respectively [22,23]. The red blood cell (RBC) samples were washed with normal saline after removing the plasma. Then, the RBC were diluted with 25x sodium phosphate buffer for superoxide dismutase (SOD) and glutathione peroxidase (GPx) measurements, and with 250x sodium phosphate buffer for catalase (CAT) measurement. The antioxidant enzymes activities (CAT, SOD, and GPx) were determined in the fresh samples and the methods for measuring these activities have been described previously [24-26]. The protein content of the plasma and RBC was determined based on the biuret reaction of the BCA kit (Thermo, Rockford, IL, USA). The values of the antioxidant enzymes activities were expressed as unit/mg of protein. All of the analyses were performed in duplicate.

### Statistical analyses

The data were analyzed using SigmaPlot software (version 12.0, Systat, San Jose, CA, USA). The normality of the distribution of the variables was evaluated using the Shapiro-Wilk test, and the normally distributed variables were age, waist circumference, the ratio of waist to hip, BMI, TC, LDL-C, HDL-C, the frequency of smoking and exercise, the ratio of vitamin E to TC, SOD, and IL-6. The multiple linear regression analyses were performed to examine the time and the interventional effects on the variables (coenzyme Q10, vitamin E, antioxidant enzymes, and inflammation markers as a dependent variable). We set the dummy variables for the time (0 = baseline, 1 = week 12) and the intervention (0 = placebo, 1 = coenzyme Q10 supplementation). If the variables had significantly effects by the time or the intervention, then the intergroup differences (intervention effect) between the placebo and the Q10-300 groups were evaluated by the Student’s t-test or the Mann-Whitney rank sum test; within a group (time effect), the paired t-test or the Wilcoxon signed rank tests were used. When comparing the changed levels of coenzyme Q10, vitamin E, antioxidant enzymes activities and inflammatory markers between the placebo and the Q10-300 groups, the Student’s t-test or the Mann-Whitney rank sum test was used. For categorical response variables, the differences between two groups were assessed by the Chi-square test (parametric method) or the Fisher’s exact test (non-parametric method). To examine the relationships of coenzyme Q10 concentration with the level of vitamin E, antioxidant enzymes activities, and inflammatory markers after the supplementation, the Pearson product moment correlations were used in the parametric data and the Spearman rank order correlations were used in the non-parametric data. The results were considered to be statistically significant at *p* < 0.05. The values presented in the text are means ± standard deviation (SD).

## Results

### Study participant characteristics

**Figure 1:**
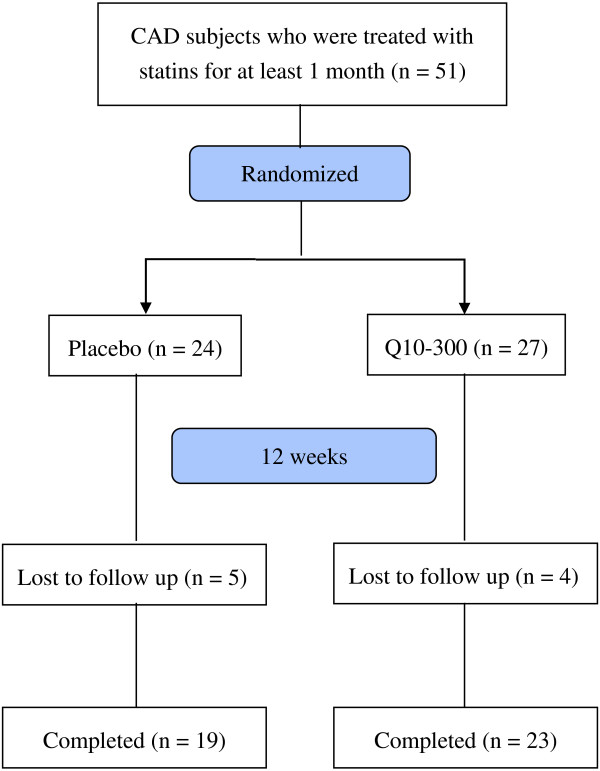
**Flow diagram.** Q10-300, coenzyme Q10 300 mg/d.

The sampling and trial profiles are summarized in Figure 1, along with the number of subjects who completed the study in each group. Table 1 shows the demographic data and the health characteristics of the subjects. There were no significant differences between the two groups with respect to age, BMI, blood pressure, anthropometric measurements, hematological entities (serum creatinine and lipid profiles), and the frequency of smoking, drinking, or exercise at baseline.

**Table 1:** Characteristics of subjects

	**Placebo (n = 19)**	**Q10-300 (n = 23)**	***P *** **values**^**1**^
Male / female (n)	12 / 7	19 / 4	0.180^a^
Age (y)	66.5 ± 11.1 (68.0)	71.7 ± 11.5 (72.0)	0.148^b^
SBP (mmHg)	133.6 ± 6.9 (130.0)	131.8 ± 12.0 (130.0)	0.175^c^
DBP (mmHg)	75.5 ± 5.2 (74.0)	73.8 ± 7.1 (70.0)	0.234^c^
Waist circumference (cm)	95.2 ± 10.0 (96.0)	92.1 ± 11.3 (90.0)	0.362^b^
Waist hip ratio	1.0 ± 0.1 (0.9)	0.9 ± 0.1 (0.9)	0.743^b^
BMI (kg/m^2^)	26.7 ± 3.2 (26.5)	25.9 ± 3.5 (25.3)	0.438^b^
Current smoker^2^, n (%)	4 (21.1%)	1 (4.3%)	0.158^d^
Drink alcohol^3^, n (%)	2 (10.5%)	3 (13.0%)	1.000^a^
Exercise^4^, n (%)	13 (68.4%)	15 (65.2%)	0.913^d^
Creatinine (μmol/L)	123.8 ± 70.7 (106.1)	114.9 ± 44.2 (97.2)	0.780^c^
TC (mmol/L)	4.5 ± 1.3 (4.4)	5.0 ± 1.0 (4.9)	0.175^b^
TG (mmol/L)	1.7 ± 1.3 (1.3)	1.6 ± 0.9 (1.4)	0.791^c^
LDL-C (mmol/L)	2.7 ± 0.8 (2.5)	3.0 ± 0.8 (3.1)	0.165^b^
HDL-C (mmol/L)	1.4 ± 0.4 (1.4)	1.4 ± 0.3 (1.4)	0.984^b^

Data are mean ± SD (median).

^1^values are significantly different between the placebo and Q10-300 groups.

^2^current smoker: individual currently smoking one or more cigarettes per day.

^3^drink alcohol: individual drinking one or more drink per day regularly.

^4^exercise: individual exercise at least 3 times every week.

^a^data were analyzed by the Fisher’s exact test.

^b^data were analyzed by the Student’s t-test.

^c^data were analyzed by the Mann-Whitney rank sum test.

^d^data were analyzed by the Chi-square test.

*BMI*, body mass index; *DBP*, diastolic blood pressure; *HDL-C*, high-density lipoprotein-cholesterol; *LDL-C*, low density lipoprotein-cholesterol; *SBP*, systolic blood pressure; *TC*, total cholesterol; *TG*, triglyceride.

### Plasma coenzyme Q10 and vitamin E concentrations

**Figure 2:**
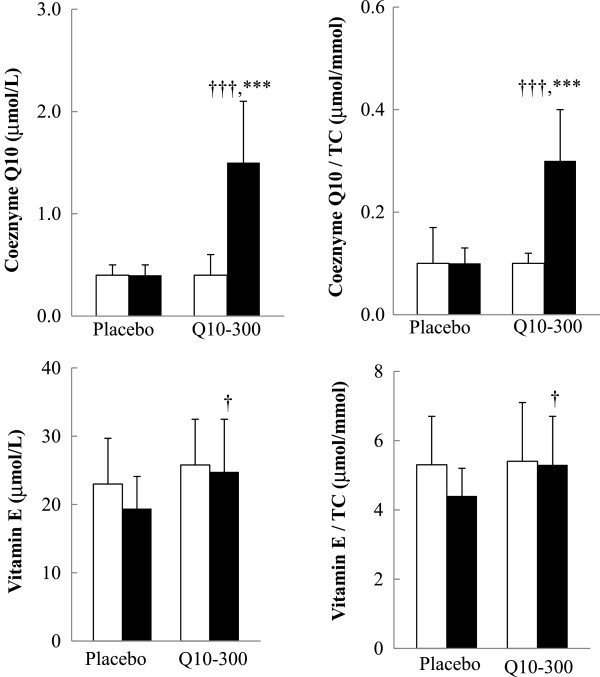
**Coenzyme Q10 and vitamin E concentrations.** Data are means ± SD. □ week 0, ■ week 12. *Values were significantly different after intervention within the group (^***^P < 0.001). †Values were significantly different between the placebo and Q10-300 groups (^†^*P* < 0.05, ^†††^*P* < 0.001). TC, total cholesterol.

The effects of coenzyme Q10 supplementation on the levels of coenzyme Q10 and vitamin E are shown in Figure 2. The plasma coenzyme Q10 concentration (*P* < 0.001) and the ratio of coenzyme Q10 to TC (*P* < 0.001) were significantly increased after coenzyme Q10 supplementation. The subjects in the Q10-300 group had significantly higher levels of coenzyme Q10 (*P* < 0.001), ratio of coenzyme Q10 to TC (*P* < 0.001), and vitamin E (*P* = 0.043), and ratio of vitamin E to TC (*P* = 0.025) than those in the placebo group at week 12.

### Antioxidant enzymes activities

**Figure 3:**
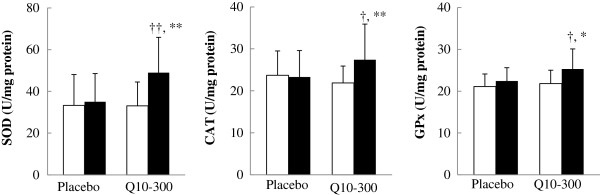
**Antioxidant enzymes activities.** Data are means ± SD. □ week 0, ■ week 12. *Values were significantly different after intervention within the group (^*^P < 0.05, ^**^P < 0.01). †Values were significantly different between the placebo and Q10-300 groups (^†^*P* < 0.05, ^††^*P* < 0.01). CAT, catalase; GPx, glutathione peroxidase; SOD, superoxide dismutase.

The effects of coenzyme Q10 supplementation on the antioxidant enzymes activities are shown in Figure 3. The activities of SOD (*P* = 0.001), CAT (*P* = 0.009), and GPx (*P* = 0.021) were significantly increased after coenzyme Q10 supplementation. The subjects in the Q10-300 group had significantly higher activities of SOD (*P* = 0.005), CAT (*P* = 0.025), and GPx (*P* = 0.040) than those in the placebo group at week 12.

### Inflammatory markers

**Figure 4:**
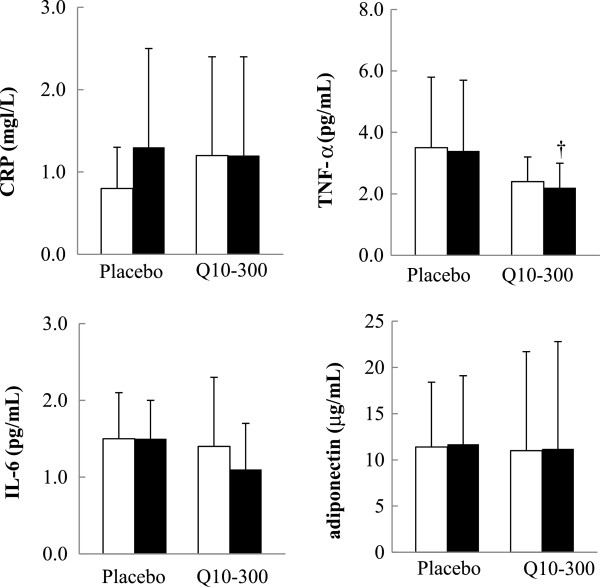
**The level of inflammatory markers.** Data are means ± SD. □ week 0, ■ week 12. †Values were significantly different between the placebo and Q10-300 groups (^†^*P* < 0.05). CRP, C-reactive protein; IL-6, interleukin-6; TNF-α, tumor necrosis factor-α.

The effects of coenzyme Q10 supplementation on the levels of inflammatory markers are shown in Figure 4. The subjects in the Q10-300 group had a significantly lower level of TNF-α than those in the placebo group at week 12 (*P* = 0.039). There were no effects on the level of CRP, IL-6, and adiponectin after 12 weeks of supplementation.

### Changed levels of coenzyme Q10, vitamin E, antioxidant enzymes activities, and inflammatory markers after supplementation

The changed levels of coenzyme Q10, vitamin E, antioxidant enzymes activities, and inflammatory markers after the supplementation are shown in Table 2. The Changed levels of coenzyme Q10 (*P* < 0.001), the ratios of coenzyme Q10 to TC (*P* < 0.001), vitamin E (*P* = 0.046), the ratios of vitamin E to TC (*P* = 0.032), antioxidant enzymes activities (SOD, *P* = 0.034; CAT, *P* = 0.033; GPx, *P* = 0.042), and inflammatory markers (TNF-α, *P* = 0.036; IL-6, *P* = 0.040) were significantly different between the placebo and the Q10-300 groups.

**Table 2:** Changed levels of coenzyme Q10, vitamin E, antioxidant enzymes activities, and inflammatory markers after supplementation

	**Placebo (n = 19)**	**Q10-300 (n = 23)**	***P *** **values**^**1**^
Coenzyme Q10 (μmol/L)	−0.01 ± 0.09 (-0.03)	1.10 ± 0.54 (1.26)	< 0.001^b^
Coenzyme Q10/TC (μmol/mmol)	−0.01 ± 0.07 (-0.00)	0.25 ± 0.11 (0.25)	< 0.001^b^
Vitamin E (μmol/L)	−3.35 ± 4.51 (-3.08)	−0.29 ± 4.03 (-0.83)	0.046^b^
Vitamin E/TC (μmol/mmol)	−0.88 ± 1.45 (-0.55)	0.07 ± 1.08 (0.11)	0.032^a^
SOD (U/mg protein)	1.63 ± 21.54 (3.63)	15.90 ± 20.48 (12.91)	0.034^a^
CAT (U/mg protein)	−1.42 ± 8.26 (-1.18)	6.17 ± 11.28 (3.18)	0.033^b^
GPx (U/mg protein)	0.91 ± 3.70 (-0.24)	4.62 ± 6.52 (2.15)	0.042^b^
CRP (mg/L)	0.39 ± 1.24 (0.00)	−0.20 ± 0.73 (0.00)	0.343^b^
TNF-α (pg/mL)	0.17 ± 0.77 (0.08)	−0.30 ± 0.35 (-0.26)	0.036^b^
IL-6 (pg/mL)	0.08 ± 0.64 (-0.11)	−0.52 ± 0.79 (-0.14)	0.040^a^
Adiponectin (μg/mL)	0.36 ± 2.89 (0.30)	0.19 ± 1.88 (-0.20)	0.71^b^

Data are mean ± SD (median).

^1^values are significantly different between the placebo and the Q10-300 groups.

^a^data were analyzed by the Student’s t-test.

^b^data were analyzed by the Mann-Whitney rank sum test.

*CAT*, catalase; *CRP*, C-reactive protein; *GPx*, glutathione peroxidase; *IL-6*, interleukin-6; *SOD*, superoxide dismutase; *TC*, total cholesterol; *TNF-α*, tumor necrosis factor-α.

### Correlations between coenzyme Q10, vitamin E, antioxidant enzymes activities, and inflammatory markers

The correlations between coenzyme Q10, vitamin E, antioxidant enzymes activities, and inflammatory markers after coenzyme Q10 supplementation are shown in Table 3. The plasma coenzyme Q10 concentration was significantly correlated with the vitamin E (*r* = 0.41, *P* = 0.008), antioxidant enzymes activities (SOD, *r* = 0.38, *P* = 0.011; CAT, *r* = 0.30, *P* = 0.0038; GPx, *r* = 0.32, *P* = 0.043), and the inflammatory markers (TNF-α, *r* = -0.33, *P* = 0.034; IL-6, *r* = -0.38, *P* = 0.027) at week 12.

**Table 3:** Correlations between coenzyme Q10, vitamin E, antioxidant enzymes activities, and inflammatory markers after coenzyme Q10 supplementation

	**Coenzyme Q10 (μmol/L) *r***^**1 **^**(*P *** **values)**
Vitamin E (μmol/L)	0.41 (0.008)^a^
SOD (unit/mg protein)	0.38 (0.011)^a^
CAT (unit/mg protein)	0.30 (0.038)^b^
GPx (unit/mg protein)	0.32 (0.043)^b^
CRP (mg/L)	−0.08 (0.621)^b^
TNF-α (pg/mL)	−0.33 (0.034)^b^
IL-6 (pg/mL)	−0.38 (0.027)^a^
Adiponectin (μg/mL)	−0.21 (0.190)^b^

^1^correlation coefficient.

^a^data were analyzed by the Pearson product moment correlation.

^b^data were analyzed by the Spearman rank order correlation.

CAT, catalase; CRP, C-reactive protein; GPx, glutathione peroxidase; IL-6, interleukin-6; SOD, superoxide dismutase; TNF-α, tumor necrosis factor-α.

## Discussion

In this clinical trial, we have demonstrated that coenzyme Q10 at the dose of 300 mg/d for 12 weeks increases the antioxidant enzymes activities (SOD, CAT, and GPx) and decreases inflammation in patients with CAD during statins therapy. Tiano et al. [27] administered coenzyme Q10 (300 mg/d) to patients with ischemic heart disease for 1 month, and they observed that those patients’ extracellular superoxide dismutase activity and endothelium-dependent vasodilatation were improved after supplementation. Antioxidant enzymes such as SOD, CAT, and GPx are the first line of defense against reactive oxygen species [28], and a decrease in their activities contributes to the elevated oxidative stress in CAD patients [14]. Our previous results showed that coenzyme Q10 at a dose of 150 mg/d increased the activity of SOD by 22.2% and of CAT by 4.5%, but had no effect on that of GPx [15]. In the present study, coenzyme Q10 supplementation at 300 mg/d increased the activity of SOD by 48.5%, CAT by 9.1%, and GPx by 4.3%, and the antioxidant enzymes activities were all positively correlated with the level of coenzyme Q10 after 12 weeks of supplementation (Table 3). It appears that the 300 mg dose of coenzyme Q10 has better antioxidation than 150 mg/d.

Both groups of statins-treated CAD patients in this study had a low level of coenzyme Q10 at baseline (Figure 2), and the level of coenzyme Q10 was significantly increased by approximately 5-fold after 4 weeks of coenzyme Q10 supplementation (data not shown; the median level of coenzyme Q10 was 0.4 to 2.0 μmol/L), which rapidly adjusted their low coenzyme Q10 level to normal values (0.5 – 1.7 μmol/L) [29]. In addition, coenzyme Q10 had a synergic effect with vitamin E [30-33]. In this study, we observed that the level of vitamin E was significantly higher in the Q10-300 group (Figure 2) and was positively correlated with the level of coenzyme Q10 after 12 weeks of supplementation (Table 3). Coenzyme Q10 not only protects vitamin E against superoxide-driven oxidation but also regenerates vitamin E during antioxidation processes [32,33].

CAD is considered to be a chronic inflammation status [10]. In the present study, the level of TNF-α (Figure 4) and the changed levels of TNF-α and IL-6 were significantly decreased after coenzyme Q10 supplementation (Table 2). The level of coenzyme Q10 was significantly negatively correlated with inflammatory markers (TNF-α and IL-6) (Table 3). Schmelzer et al. [34,35] demonstrated that coenzyme Q10 could exert anti-inflammation effects via the reduction of nuclear factor-κB (NF-κB) dependent gene expression. NF-κB can be activated by the reactive oxygen species and can then up-regulate pro-inflammatory cytokines expression. However, this NF-κB -activating cascade could be inhibited by antioxidants, such as coenzyme Q10 [36]. Our previous results [21] showed that coenzyme Q10 at a dose of 150 mg/d decreased the level of IL-6 by 0.40 pg/mL. In the present study, coenzyme Q10 supplementation at 300 mg/d decreased the levels of TNF-α by 0.30 pg/mL and IL-6 by 0.52 pg/mL. However, coenzyme Q10 supplementation had no effect on the level of CRP. As we previously reported, proinflammatory cytokines (TNF-α and IL-6) reflect the status of inflammatory reactions with more sensitivity (easily changed) than CRP, which is a product of hepatic stimulation [21]. A higher level of adiponectin is associated with a lower risk of CAD [20,37], but coenzyme Q10 supplementation had no effect on the level of adiponectin in the present study. Nakamura et al. [20] reported that the concentration of plasma adiponectin was not significantly different between stable angina pectoris and control subjects. The subjects who had CAD in this study had not experienced acute myocardial infarction within the previous 6 months and thus were stable angina pectoris patients, which might be the reason why we did not find an effect of coenzyme Q10 on adiponectin.

Regarding the safety of statins therapy and its combination with placebo or coenzyme Q10 in the present study, there were no clinically significant changes in the subjects’ vital signs, serum chemical values, or hematological values, and there were no serious adverse events, no complaints of myalgia or muscle weakness, and no withdrawals due to adverse events. Thus, coenzyme Q10 at a dose of 300 mg/d is safe for co-administration with statins therapy.

Two limitations of the present study should be mentioned. First, the number of participants was small, although we did recruit more subjects than expected. Second, a few of the subjects had higher inflammation in the present study. Only 10% of the subjects had a high inflammation according to the level of CRP (≥ 3.0 mg/L), and this finding might have contributed to observing the null effect on CRP after the supplementation. Large studies are needed to establish the beneficial effect of coenzyme Q10 supplementation on inflammation, especially in subjects who have high inflammation status.

## Conclusions

In conclusion, we have demonstrated that coenzyme Q10 supplementation at a dose of 300 mg/d significantly increased antioxidant enzymes activities and reduced the levels of inflammatory markers (TNF-α and IL-6) in CAD patients during statins therapy. CAD patients might benefit from using coenzyme Q10 supplements to increase their antioxidation and anti-inflammation capacity during statins therapy.

## Competing interests

The authors have no conflict of interest.

## Authors’ contributions

BJL carried out the study, performed the data analyses, and drafted the manuscript. YFT and CHY carried out the study and sample analyses. PTL conceived of the study, participated in its design, and coordination, and helped to draft the manuscript. All authors read and approved the final manuscript.
